# Temperature analysis of 3D-printed biomaterials during unipolar and bipolar radiofrequency ablation procedure

**DOI:** 10.3389/fcvm.2022.978333

**Published:** 2022-09-14

**Authors:** Ida Anna Cappello, Mara Candelari, Luigi Pannone, Cinzia Monaco, Edoardo Bori, Giacomo Talevi, Robbert Ramak, Mark La Meir, Ali Gharaviri, Gian Battista Chierchia, Bernardo Innocenti, Carlo de Asmundis

**Affiliations:** ^1^Heart Rhythm Management Centre, Postgraduate Program in Cardiac Electrophysiology and Pacing, Universitair Ziekenhuis Brussel - Vrije Universiteit Brussel, European Reference Networks Guard-Heart, Brussels, Belgium; ^2^BEAMS Department (Bio Electro and Mechanical Systems), Université Libre de Bruxelles, Brussels, Belgium; ^3^Cardiac Surgery Department, Universitair Ziekenhuis Brussel - Vrije Universiteit Brussel, Brussels, Belgium

**Keywords:** biomaterial tests, thermal test, radiofrequency ablation, 3D surgical guide, arrhythmias treatment

## Abstract

**Background:**

Due to their mechanical properties, the MED625FLX and TPU95A could be appropriate candidates for cardiac 3D surgical guide use during radiofrequency ablation (RFA) treatment.

**Methods:**

RFA aims to destroy the heart tissue, which cause arrhythmias, by applying a radiofrequency (RF) energy at critical temperature above +50.0°C, where the thermal damage is considered not reversible. This study aims to analyze the biomaterials thermal properties with different thicknesses, by testing the response to bipolar and unipolar RFA on porcine muscle samples (PMS), expressed in temperature. For the materials evaluation, the tissue temperature during RFA applications was recorded, firstly without (control) and after with the biomaterials in position. The biomaterials were considered suitable for the RFA treatment if: (1) the PMS temperatures with the samples were not statistically different compared with the control; (2) the temperatures never reached the threshold; (3) no geometrical changes after RFA were observed.

**Results:**

Based on these criteria, none of the tested biomaterials resulted appropriate for unipolar RFA and the TPU95A failed almost all thermal tests also with the bipolar RFA. The 1.0 mm MED625FLX was modified by bipolar RFA in shape, losing its function. Instead, the 2.5 mm MED625FLX was considered suitable for bipolar RFA catheter use only.

**Conclusions:**

The 2.5 mm MED625FLX could be used, in the design of surgical guides for RFA bipolar catheter only, because of mechanical, geometrical, and thermal properties. None of biomaterials tested are appropriate for unipolar ablation catheter because of temperature concerns. Further investigations for clinical use are eagerly awaited.

## Introduction

The MED625FLX and TPU95A are two biomaterials suitable for 3D printing technology in the medical field, due to their biocompatible properties (1). In detail, MED625FLX is a polymeric material, characterized by transparence, flexibility, biocompatibility and certified for bodily contact. The material is already approved for dental use for permanent skin contact (more than 30 days) and up to 24 h mucosal membrane contact, including indirect bonding trays and soft gingiva masks. However, there are no data about internal tissue, like cardiac myocardium. Nevertheless, for an initial biological risks evaluation, some biological tests were performed on MED625FLX according to the International Organization for Standardization (ISO) requirements (2). Instead, the thermoplastic polyurethane (TPU) is a polymeric material that can be manipulated, molded, and produced through heating in various industrial processes. It is composed of three materials: a diisocyanate, a chain extender and a macrodiol (or polyol) which are linked to form linear, segmented copolymers consisting of alternating hard and soft segments. Due to the soft to hard segments, TPU exhibits a broad range of mechanical properties, across a wide range of temperatures, resulting in excellent physical properties and biocompatibility (3). TPU95A tests to evaluate mechanical properties were experimentally executed by Ultimaker (4). Thus, it is widely used in biomedical environment for vascular catheters, blood bags, implants targeting both soft and hard tissues and, other medical device components (5). Among the different surgical and therapeutic procedures used in the cardiac field, radiofrequency ablation (RFA) is an effective therapeutic intervention in patients with arrhythmias. It consists of destroying the heart tissue which causes the arrhythmia by heat, applying a high frequency electrical energy, such as radiofrequency (RF) to a desired arrhythmogenic site in the patient's heart. When RF energy is delivered, the current propagates radially from the source and the current density decreases in proportion to the square of the distance from the RF electrode source. Thus, only the narrow part of the tissue in close contact with the catheter electrode is heated directly. In the deeper tissue layers the heating occurs passively through heat conduction. If higher power is used, both the depth of direct resistive heating and the volume and radius of the virtual heat source will increase. Lesion dimensions also depend on the ablation electrode diameter (6). RF generator usually provides a continuous unmodulated sine wave output in the frequency range of 300 kHz to 1 MHz to the metal electrode, causing a temperature increase both in the surrounding tissue and in the tip. The thermal damage is considered not reversible if the tissue temperature raises at values above +50.0°C, because the threshold for thermal damage is demonstrated to be set at +43.0°C (7).

Therefore, this study aims to perform additional thermal tests, analyzing the materials behavior in response to RFA, as there is a lack of data in literature on this topic. Moreover, no previous studies have been performed for thermal evaluation of 3D printable biomaterials in relation to unipolar and bipolar radiofrequency energy. This is propaedeutic to their application, for a 3D surgical guides, during cardiac ablation treatment. In particular, the aim of the study is to investigate MED625FLX and TPU95A for any mechanical or geometric alterations during RFA. Furthermore, the behavior of the materials as thermal insulants or as conductors is assessed.

## Methods

In this study, two different ablation catheters and RFA modality were analyzed: unipolar and bipolar RF catheters. With bipolar ablation, RF current flows between two catheter electrodes. In contrast, with unipolar ablation, RF current flows between the tip of the ablation catheter electrode and the indifferent electrode of the ground patch. They differ in type lesion performed: lesion depth is significantly greater with bipolar compared to unipolar (8).

For this study, the AtriCure Coolrail Linear Pen (Coolrail; Atricure Inc, USA), was selected as bipolar catheter, delivering bipolar RF energy, which flows between the linear electrodes parallelly placed on the catheter head. The Coolrail Linear Pen is an irrigated radiofrequency probe with two linear electrodes of 30.0 mm in length; the head can rotate for a total articulation of 50.0°, to access difficult anatomies, allowing angles promoting consistent tissue contact during ablation. According to clinical evaluations and instructions for use (IFU), the ablation catheter was set at +70 W and +70.0°C for a RFA application of 30.0 s (9). As unipolar catheter, the TactiCath Contact Force Ablation Catheter (Abbott, Chicago, USA), a 4-mm irrigated catheter, was chosen to perform the thermal tests. This includes the 3.5 mm tip electrode with 6-holes irrigation and the distal deformable body for contact force sensing. The thermocouple sits at 2.67 mm from the distal end of the tip electrode, while the ring space between electrodes is 2.0 mm. It was set at +50 W and + 70.0°C for a RFA of 60 s, based on IFU and clinical assessment (10).

Porcine muscle samples (PMS), with a thickness of 10.0 mm, were used and analyzed for the test; the reason is that the surgical guide is aimed for ventricles and the porcine muscle thickness is similar to human ventricular wall (11). Moreover, to reproduce the human physiological conditions, the PMS were kept in a Plexiglass box filled of water at +37.0°C (body temperature).

No need for approval from the Institutional Animal Welfare Committee about *ex vivo* tissue utilization was required.

### Printing parameters

For the purpose of this work, the MED625FLX and TPU95A were the object of study. All 3D printed objects for RFA were manufactured according to ISO standards.

The transparent and flexible MED625FLX was printed by Objet260 Connex1 3D printer (Stratasys Ltd), using Polyjet technology. The MED625FLX semicircular samples were printed in single material mode and the printer was set for glossy printing, in order to obtain a more resistant layer. The thickness of each layer measured 16.0 nanometers; the build mode was set in high speed (0.001 in. resolution).

Concerning the TPU95A, the Anycubic Mega Zero 2.0 3D printer (Shenzhen Anycubic Technology Co., Ltd) was used to realize the samples to test, based on Fused Deposition Modeling (FDM), an additive manufacturing process that belongs to the material extrusion family. In FDM, an object is built by selectively depositing melted material in a pre-determined path layer-by-layer. It can print at a minimum layer height of 100.0 microns and the print speed was automatically set at 60.0 mm/s. To make the 3D printer compatible with TPU95A, the machine's extruder temperature was set at 205.0°C, whereas the heated bed temperature of 110.0°C (12).

Due to the absence of manufacturer data on the MED625FLX, we have assumed thermal properties of MED625FLX similar to rubber. The thermal conductibility of rubber is K_med_=0,4 W/m^*^K (13). The thermal conductibility of TPU95A is K_tpu_=0,15 W/m^*^K (14).

### Experimental parameters

**Figure 1:**
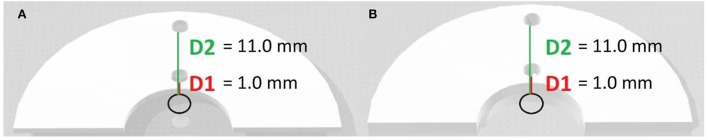
3D models of biomaterial samples. 3D models of different thickness of semicircular-shaped samples on which the experimental tests were performed. **(A)** 1.0 mm of thickness; **(B)** 2.5 mm of thickness.

**Figure 2:**
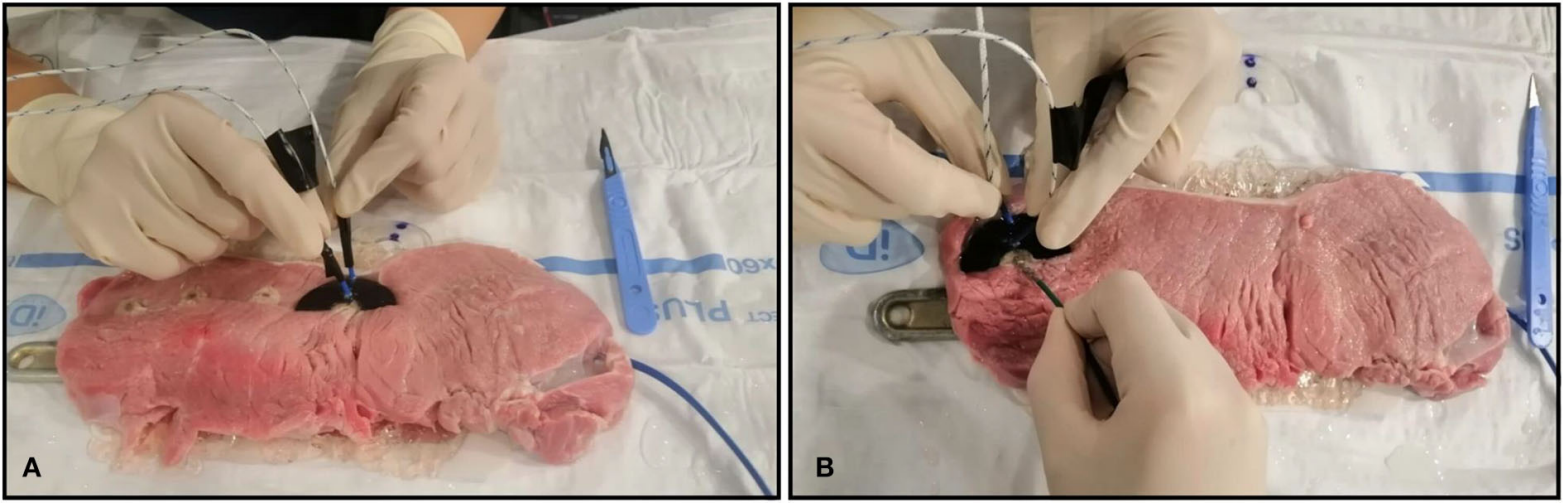
*Ex vivo* experimental setting. *Ex vivo* simulation of radiofrequency ablation (RFA). **(A)** The J type thermocouples positioned inside the sample D_1_ and D_2_ holes; **(B)** Example of unipolar RFA on TPU95A of 2.5 mm.

The experimental tests were performed on 8 printed samples consisting in semicircular-shaped samples, with an internal diameter of 15.0 mm and an external diameter of 45.0 mm (Figure 1). Also, different thicknesses were analyzed in order to assess if the materials could act as insulants or as conductors. In Figure 1 the black circles represent the point of RFA application, D_1_ represents the distance of 1.0 mm from the RFA source, and D_2_ the distance of 11.0 mm from RFA source. The holes in D_1_ and D_2_ were realized to insert two thermocouples, to measure the underlying tissue temperature in the two spots at 1.0 mm and 11.0 mm of distance from the RFA source, simultaneously. The thermometer used was the VOLTCRAFT PL-125-T4 with a sensor type K, a tip of 4.0 mm, a temperature range between −200.0°C and +1,372.0°C and an accuracy of ± 1.0°C (15). The Figure 2A. shows how the thermocouples were positioned inside the sample holes D_1_ and D_2_. The Figure 2B. shows an example of a unipolar RFA on a TPU95A sample of 2.5 mm. Since the energy propagation and the lesion dimensions are not only directly proportional to the power, temperature delivered and the radiofrequency time, but also inversely proportional the distance from the source, the temperature analysis was performed at D_1_ and at D_2_, to verify if the material alters the energy propagation. For the experimental purpose, 2.5 mm and 1.0 mm material sample thickness were examined, considering that a higher thickness makes the material stiffer (Figure 1).

In addition, as the MED625FLX and TPU95A were thought for medical use, and thus they could be placed in contact with biological tissues, an efficient sterilization is crucial to minimize the risk of infections, which are a major concern in health care. However, the sterilization method, a high temperature process, could alter the mechanical and thermal properties of the material itself. Therefore, before testing, the samples were cleaned in a specific washing machine at +93.0°C for 1 h and 30 min, and then, the samples were subjected to the Sterrad Vaporized Hydrogen Peroxide (VHP) Gas Sterilization. Subsequently, they were placed into VHP sterilization machine (Sterrad Sterilization System of Johnson & Johnson) at +45.0°C for 40 min (16).

Furthermore, to evaluate any changes in energy (heat) propagation on the tissue underneath the samples, depending on sample thickness, two thermocouple probes were inserted in the underlying tissue through holes at specific distances D_1_ and D_2_ from the RF source, as shown in Figure 2A.

For the materials evaluation, the tissue temperature behavior was recorded during RFA procedure, firstly without (control) and after with the sample in position. For the temperature measurements, to evaluate the role of repetitive RFA applications, each material sample underwent to four consecutive applications. Temperatures were measured at two distances D_1_ and D_2_. Exact time references were used, and temperature measured accordingly during continuous RFA: 0, 10, 15, and 30 s for bipolar catheter and 0, 10, 15, 30, and 60 s for unipolar catheter.

During the assessment, the material samples were placed on PMS, and they underwent to four ablation sessions; the temperature measurements were recorded in D_1_ and D_2_ at each time instant previously defined. The ablation was delivered each time in different PMS position in order to avoid that the new measurements depended on the previous ablations. At the end of each session of 4 RFA applications, the biomaterial samples of MED625FLX and TPU95A of both thicknesses, were placed on a grid paper, to examine any eventual alterations in shape due to the RFA. The whole procedure was performed firstly by delivering RF from bipolar catheter and then from the unipolar one.

Therefore, by the experimental trials, the materials will be considered suitable for the RFA treatment whether they satisfy the following requirements:

- The increased energy propagation in the underlying tissue is not statistically significant with respect to the heat propagation in the control tissue.
- During the experimental test, the underlying temperatures never reach the threshold, set at +43.0°C, as previously explained.
- No observable geometrical changes after RFA applications.

### Statistical analysis

Statistical analysis was performed in order to compare temperatures reached by the tissue with and without MED625FLX and TPU95A samples, in response to both bipolar and unipolar ablation catheters. Measurements were repeated at specific time instants, by distinguishing different thicknesses and different distances D_1_ and D_2_. All variables were tested for normality with Shapiro–Wilk test. Normally distributed variables were described as mean ± standard deviation and the groups were compared through unpaired *t*-test as appropriate, while the non-normally distributed variables were described as median (Inter Quartile Range) and compared by Mann-Whitney test or Wilcoxon signed-rank test as appropriate. The categorical variables were described as frequencies (percentages) and compared by Chi-squared test or Fisher's exact test as appropriate.

A *p*-value < 0.05 was considered statistically significant. The analysis was performed using R software version 3.6.2 (R Foundation for Statistical Computing, Vienna, Austria).

## Results

The results of temperature changes at D_1_ and D_2_ in response to 30 s of four RFA applications by bipolar catheter and in response to 60 s of four RFA applications by unipolar catheter, without the samples (control) are summarized in Table 1.

**Table 1:** Temperatures' measurement of the control during RFA performed by the bipolar and unipolar.

**BipolarCatheter**	Temperature in D_**1**_ (**°**C)					Temperature in D_**2**_ (**°**C)				
Control^*^	34.4	34.5	34.5	34.7	–	–	29.7	29.7	29.8	–
Control^**^	27.9	28.2	28.2	28.7	–	27.0	27.0	27.1	27.2	–
Control^***^	32.5	32.9	33.1	33.7	–	30.5	31.1	31.3	31.6	–
Control^****^	30.9	31.2	31.4	31.6	–	30.1	30.2	30.2	30.5	–
Time	0 s	10 s	15 s	30 s	–	0 s	10 s	15 s	30 s	–
**UnipolarCatheter**	**Temperature in D**_1_ **(**°**C)**					**Temperature in D**_2_ **(**°**C)**				
Control^*^	36.3	38.3	38.4	38.2	–	30.1	30.8	31.4	31.1	–
Control^**^	35.8	37.5	37.9	38.4	–	31.6	33.2	33.8	34.9	–
Control^***^	34.0	35.9	36.3	36.6	37.2	32.2	33.4	34.4	35.3	36.8
Control^****^	32.6	33.6	33.8	34.5	35.2	30.5	31.1	31.1	31.4	32.7
Time	0 s	10 s	15 s	30 s	60 s	0 s	10 s	15 s	30 s	60 s

The control refers to the temperatures' measurement of PMS during RFA performed by the bipolar and unipolar catheters without the biomaterials in place at D_1_ (1.0 mm) and D2 (11.0 mm). Each star (^*^) identifies the number of ablations performed.

The temperature assessment with the material samples in position (MED625FLX and TPU95A, 2.5 mm of thickness), at distances D_1_ and D_2_ from RFA with both bi- and uni-polar catheters is summarized in Tables 2, 3. Instead, the temperature measurements, at distance D_1_ and D_2_ from both bi- and uni-polar catheters with 1.0 mm MED625FLX and 1.0 mm TPU95A samples in position, are presented in Tables 4, 5.

**Table 2:** Temperature measurements under the MED625FLX samples of 2.5 mm during bipolar and unipolar RFA catheter.

**Bipolar catheter**										
**MED625FLX 2.5 mm**	**Temperature in D**_**1**_ **(°C)**					**Temperature in D**_**2**_ **(°C)**				
1^*^	31.8	32.8	32.8	33.9	–	29.2	29.2	29.2	29.2	–
1^**^	33.4	33.8	33.9	34.5	–	29.6	29.4	29.5	29.7	–
1^***^	35.0	36.2	36.2	37.1	–	29.7	29.8	29.8	29.8	–
1^****^	32.3	32.8	33.4	33.7	–	30.3	30.5	30.6	30.9	–
Time	0 s	10 s	15 s	30 s	–	0 s	10 s	15 s	30 s	–
**Unipolar catheter**										
**MED625FLX 2.5 mm**	**Temperature in D**_1_ **(**°**C)**					**Temperature in D**_2_ **(**°**C)**				
10^*^	44.5	45.6	45.0	42.3	38.9	35.3	32.1	31.6	31.1	30.8
10^**^	50.6	46.0	44.3	42.1	39.3	34.4	32.5	31.7	30.8	30.7
10^***^	75.2	65.4	60.0	52.9	45.2	31.8	31.9	32.0	32.3	33.0
10^****^	66.0	45.5	48.8	46.2	43.0	33.1	33.3	33.2	33.1	33.2
Time	0 s	10 s	15 s	30 s	60 s	0 s	10 s	15 s	30 s	60 s

The 2.5 mm of MED625FLX samples are tested during RFA procedure performed by bi- and uni-polar catheters. The temperatures of underlying tissue were measured at different distances D_1_ (1.0 mm) and D2 (11.0 mm) from the source at 0, 10, at 15, 30, and 60 s after ablation. Each star (^*^) identifies the number of ablations performed.

**Table 3:** Temperature measurements under the TPU95A samples of 2.5 mm during bipolar and unipolar RFA catheter.

**Bipolar catheter**										
**TPU95A 2.5 mm**	**Temperature in D**_**1**_ **(°C)**					**Temperature in D**_**2**_ **(°C)**				
2^*^	31.1	32.0	32.1	32.6	–	28.0	27.9	28.1	28.4	–
2^**^	32.9	33.3	33.4	34.0	–	30.4	30.4	30.6	30.4	–
2^***^	37.0	39.8	39.9	40.0	–	30.8	30.8	30.4	31.2	–
2^****^	44.0	43.2	42.1	41.0	–	29.8	29.8	30.1	30.5	–
Time	0 s	10 s	15 s	30 s	–	0 s	10 s	15 s	30 s	–
**Unipolar catheter**										
**TPU95A 2.5 mm**	**Temperature in D**_1_ **(**°**C)**					**Temperature in D**_2_ **(**°**C)**				
20^*^	66.3	54.5	52.4	48.8	44.0	36.2	34.7	34.5	34.4	34.2
20^**^	65.8	57.6	55.4	50.7	44.2	34.2	31.6	30.1	30.1	30.3
20^***^	71.1	58.0	56.2	51.8	45.8	45.5	37.6	37.0	35.0	34.3
20^****^	56.0	53.0	52.2	49.2	44.2	29.4	31.1	30.9	31.1	32.3
Time	0 s	10 s	15 s	30 s	60 s	0 s	10 s	15 s	30 s	60 s

The 2.5 of TPU95A samples are tested during RFA bi- and uni-polar procedure. The temperatures of underlying tissue were measured at different distances D_1_ (1.0 mm) and D_2_ (11.0 mm) from the source at 0, 10, at 15, 30, and 60 s after ablation. Each star (^*^) identifies the number of ablations performed.

**Table 4:** Temperature measurements under the MED625FLX samples of 1.0 mm during bipolar and unipolar RFA catheter.

**Bipolar catheter**										
**MED625FLX 1.0 mm**	**Temperature in D**_**1**_ **(°C)**					**Temperature in D**_**2**_ **(°C)**				
3^*^	33.2	33.5	33.7	34.1	–	32.5	32.5	32.6	32.6	–
3^**^	32.4	33.0	33.3	33.9	–	32.1	32.2	32.2	32.1	–
3^***^	33.3	33.1	33.7	34.0	–	32.3	32.1	32.1	32.3	–
3^****^	37.8	40.0	39.8	38.4	–	31.1	31.0	31.0	31.1	–
Time	0 s	10 s	15 s	30 s	–	0 s	10 s	15 s	30 s	–
**Unipolar catheter**										
**MED625FLX 1.0 mm**	**Temperature in D**_1_ **(**°**C)**					**Temperature in D**_2_ **(**°**C)**				
30^*^	39.2	39.4	39.6	39.4	38.6	34.5	33.8	33.7	33.3	33.1
30^**^	33.8	35.2	35.5	35.8	36.7	33.6	33.4	33.3	34.0	34.6
30^***^	60.0	48.7	47.0	44.5	41.9	35.1	34.4	34.0	34.1	34.1
30^****^	68.0	55.0	51.7	47.0	42.2	37.3	36.8	36.5	36.5	36.5
Time	0 s	10 s	15 s	30 s	60 s	0 s	10 s	15 s	30 s	60 s

The 1.0 mm of MED625FLX samples are tested during RFA procedure performed by bi- and uni-polar catheter. The temperatures of underlying tissue were measured at different distances D_1_ (1.0 mm) and D_2_ (11.0 mm) from the source at specific time instants after ablation. Each star (^*^) identifies the number of ablations performed.

**Table 5:** Temperature measurements under the TPU95A samples of 1.0 mm during bipolar and unipolar RFA catheter.

**Bipolar catheter**										
**TPU95A 1.0 mm**	**Temperature in D**_**1**_ **(°C)**					**Temperature in D**_**2**_ **(°C)**				
4^*^	32.6	32.8	32.7	33.0	–	32.2	32.2	32.2	32.1	–
4^**^	32.2	32.5	32.6	33.0	–	31.8	31.8	31.7	31.7	–
4^***^	39.2	39.6	40.1	40.3	–	32.3	31.9	31.9	31.6	–
4^****^	49.5	47.6	46.6	44.3	–	32.8	32.3	32.2	32.2	–
Time	0 s	10 s	15 s	30 s	–	0 s	10 s	15 s	30 s	–
**Unipolar catheter**										
**TPU95A 1.0 mm**	**Temperature in D**_1_ **(**°**C)**					**Temperature in D**_2_ **(**°**C)**				
40^*^	35.3	35.6	36.4	37.1	38.3	34.1	34.2	34.6	35.2	36.5
40^**^	48.6	46.3	46.1	45.4	43.0	35.3	35.6	36.5	37.3	37.6
40^***^	73.6	63.5	59.0	52.0	46.2	36.7	36.6	36.6	37.0	36.8
40^****^	66.4	60.8	59.2	54.5	47.0	29.5	30.5	30.5	31.2	32.5
Time	0 s	10 s	15 s	30 s	60 s	0 s	10 s	15 s	30 s	60 s

The 1.0 mm of TPU95A samples are tested during RFA procedure performed by bi- and uni-polar catheter. The temperatures of underlying tissue were measured at different distances D_1_ (1.0 mm) and D_2_ (11.0 mm) from the source at specific time instants after ablation. Each star (^*^) identifies the number of ablations performed.

The results, obtained by bipolar catheter application with MED625FLX and TPU95A 2.5 mm samples in position, showed that, at each time instant, the temperature differences were not statistically significant compared with control temperature, in both D_1_ and D_2_ (Supplementary Tables 1, 2).

Different results were obtained during unipolar RFA applications with MED625FLX and TPU95A 2.5 mm samples; indeed, at each time instant, the temperature differences were statistically significant compared with control temperature in D_1_ for both MED625FLX and TPU95A 2.5 mm (Table 6, Supplementary Table 3). There was no statistical difference between temperatures with and without material samples in D_2_, except for the MED625FLX 2.5 mm at 0 s (*p* = 0.030) (Supplementary Table 4).

**Table 6:** Statistical Analysis between control and PMS under the biomaterials in D_1_ with unipolar catheter.

**Unipolar catheter in D**_**1**_				
**Time**	**Biomaterial**	**Control mean (SD)**	**Biomaterial mean (SD)**	***P*** **-value**
0 s	Med_2.5 mm	34.7 (1.7)	59.1 (14.1)	0.014
10 s	Med_2.5 mm	36.3 (2.1)	50.6 (9.9)	0.030
15 s	Med_2.5 mm	36.6 (2.1)	49.5 (7.3)	0.014
30 s	Med_2.5 mm	36.9 (1.8)	45.9 (5.0)	0.016
60 s	Med_2.5 mm	36.2 (1.4)	41.6 (3.0)	0.083
0 s	Tpu_2.5 mm	34.7 (1.7)	64.8 (6.3)	<0.001
10 s	Tpu_2.5 mm	36.3 (2.1)	55.8 (2.4)	<0.001
15 s	Tpu_2.5 mm	36.6 (2.1)	54.0 (2.0)	<0.001
30 s	Tpu_2.5 mm	36.9 (1.8)	50.1 (1.4)	<0.001
60 s	Tpu_2.5 mm	36.2 (1.4)	44.5 (0.8)	<0.001

For the *p*-value computation different thicknesses of biomaterials have been considered. The SD is the standard deviation of temperature measurements.

The temperature tests with bipolar RFA for MED625FLX and TPU95A 1.0 mm samples in position, demonstrated no differences in D_1_ compared with control (Supplementary Table 1); while in D_2_ the temperature change was statically significant only for MED625FLX and TPU95A 1.0 mm sample at 0, 10, and 15 s (Table 7, Supplementary Table 2).

**Table 7:** Statistical Analysis between control and PMS under the biomaterials in D_2_ with bipolar catheter.

**Bipolar catheter in D2**				
**1-5 Time**	**Biomaterial**	**Control mean (SD)**	**Biomaterial mean (SD)**	***P*** **-value**
0 s	Med_1.0 mm	29.2 (1.9)	32.0 (0.6)	0.037
10 s	Med_1.0 mm	29.5 (1.8)	32.0 (0.7)	0.040
15 s	Med_1.0 mm	29.6 (1.8)	32.0 (0.7)	0.046
30 s	Med_1.0 mm	29.8 (1.9)	32.0 (0.6)	0.063
0 s	Tpu_1.0 mm	29.2 (1.9)	32.3 (0.4)	0.024
10 s	Tpu_1.0 mm	29.5 (1.8)	32.0 (0.2)	0.029
15 s	Tpu_1.0 mm	29.6 (1.8)	32.0 (0.2)	0.036
30 s	Tpu_1.0 mm	29.8 (1.9)	31.9 (0.3)	0.066

For the *p*-value computation different thicknesses of biomaterials have been considered. The SD is the standard deviation of temperature measurements.

The assessment of MED625FLX and TPU95A 1.0 mm samples with unipolar RFA, showed that there was no significant difference between temperatures in D_1_, except for the sample of TPU95A 1.0 mm at 30 s (*p* = 0.041) (Supplementary Table 3); in D_2_ there was no significant statistical difference in temperatures, except for the sample of MED625FLX 1.0 mm at 0 s (*p* = 0.005) and at 10 s (*p* = 0.046) (Supplementary Table 4).

**Figure 3:**
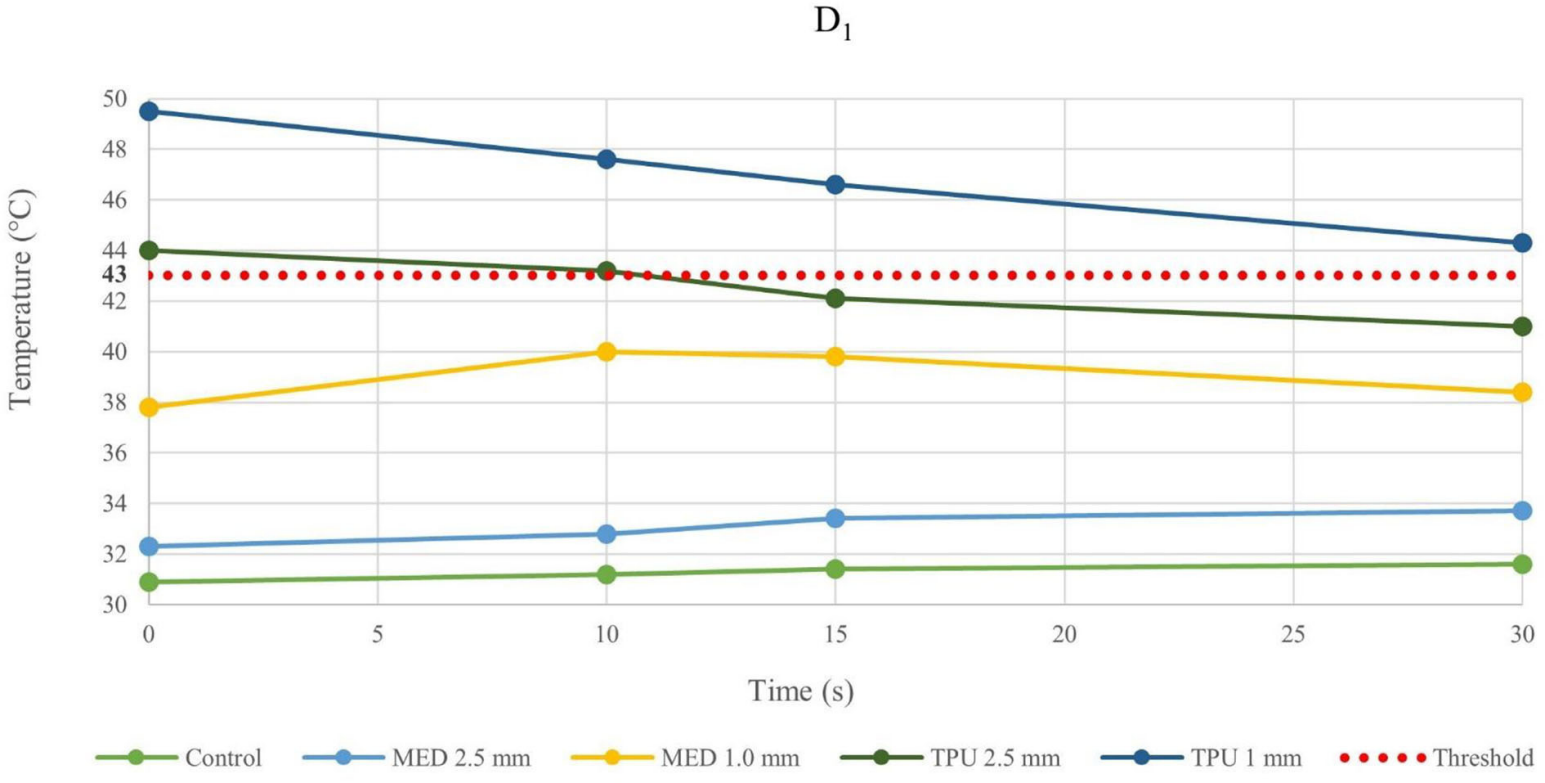
Temperature behavior of control and biomaterial samples performed by bipolar catheter in D_1_. The temperature behavior of the control, the MED625FLX and the TPU95A of 1.0 mm and 2.5 mm at D_1_ due to RFA performed by bipolar catheter; the dashed line indicates the threshold set at +43.0°C.

**Figure 4:**
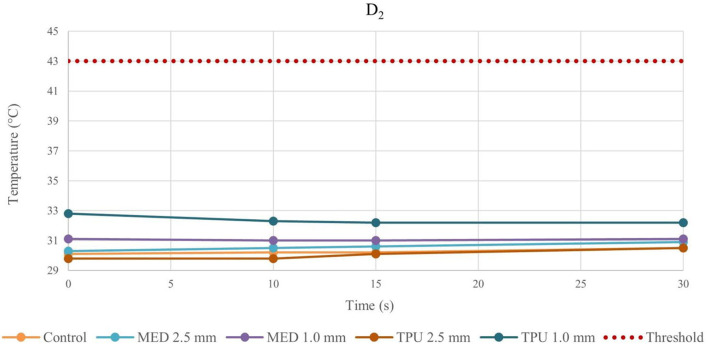
Temperature behavior of control and biomaterial samples performed by bipolar catheter in D_2_. The temperature behavior of the control, the MED625FLX and the TPU95A of 1.0 mm and 2.5 mm at D_2_ due to RFA performed by bipolar catheter; the dashed line indicates the threshold set at +43.0°C.

**Figure 5:**
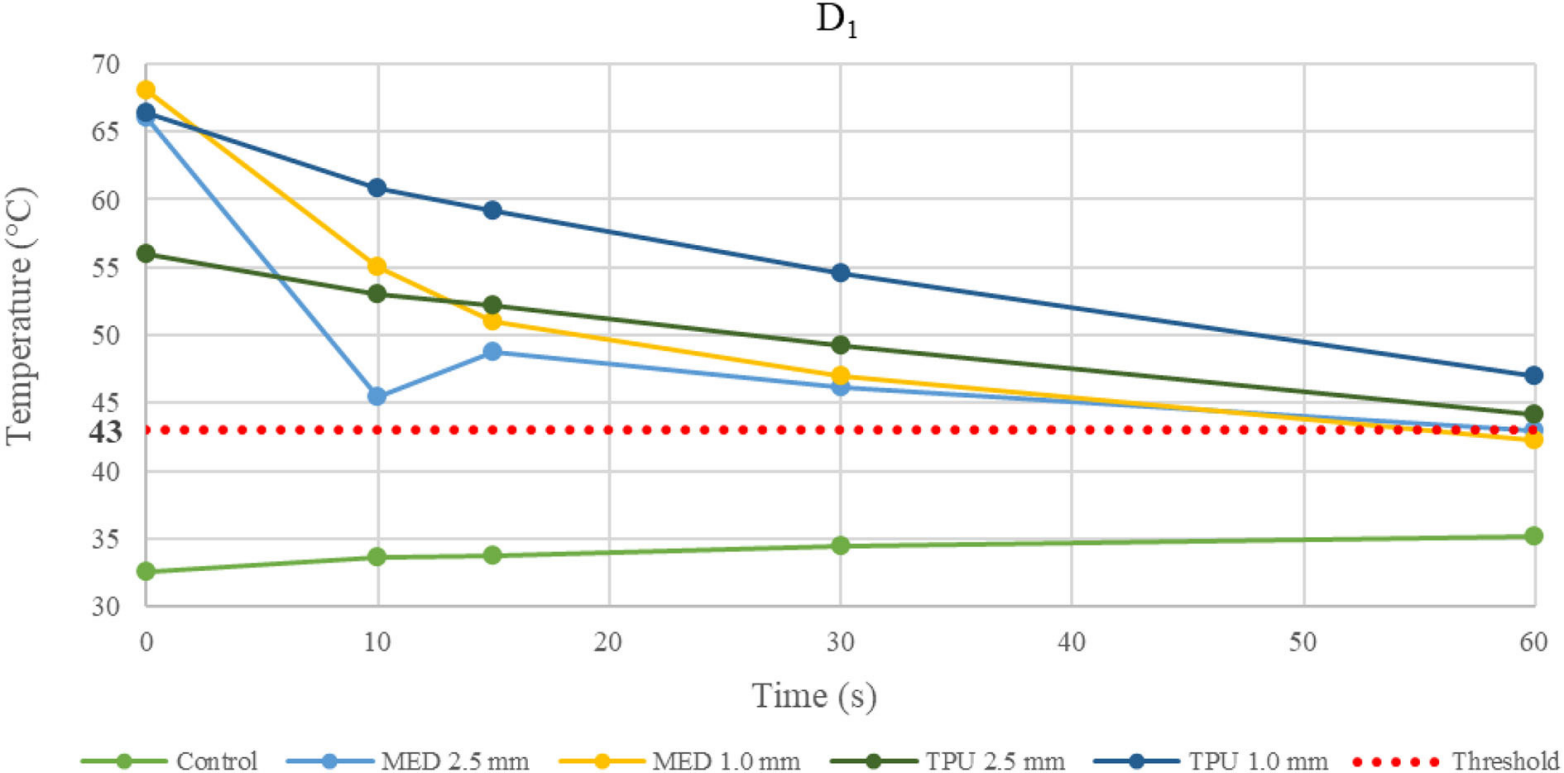
Temperature behavior of control and biomaterial samples performed by unipolar catheter in D_1._ The temperature behavior of the control, the MED625FLX and the TPU95A of 1.0 mm and 2.5 mm at D_1_ due to RFA performed by unipolar catheter; the dashed line indicates the threshold set at +43.0°C.

**Figure 6:**
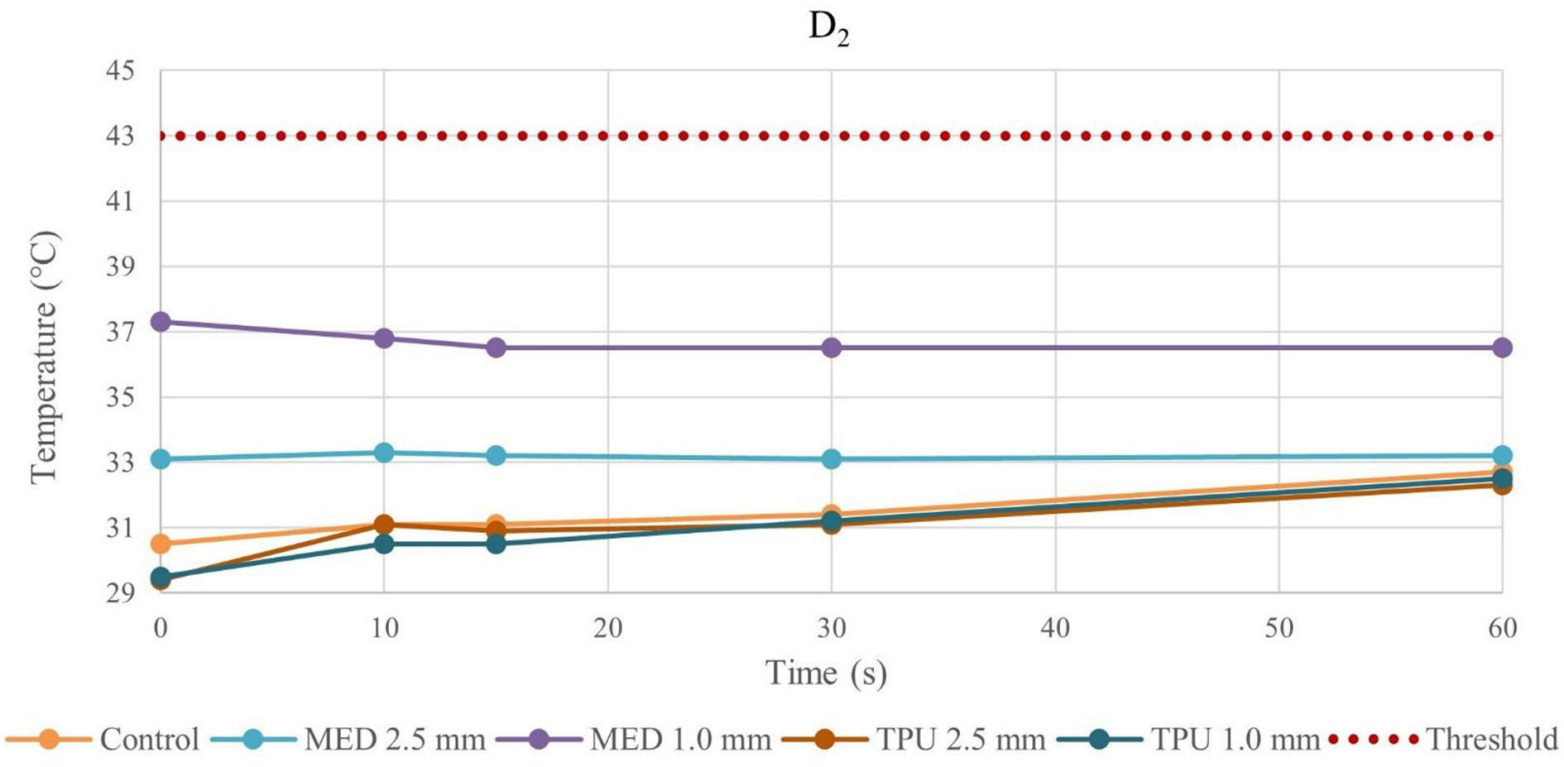
Temperature behavior of control and biomaterial samples performed by unipolar catheter in D_2_. The temperature behavior of the control, the MED625FLX and the TPU95A of 1.0 mm and 2.5 mm at D_2_ due to RFA performed by unipolar catheter; the dashed line indicates the threshold set at +43.0°C.

Because of the material stress due to RFA, the tissue temperatures after the fourth application were examined in detail and reported in the line charts, illustrating the temperatures evolution in time of the fourth RFA application of the control and material samples for both procedures performed by bipolar catheter for distance D_1_ (Figure 3) and distance D_2_ (Figure 4); the temperature values at D_1_ and D_2_ from unipolar catheter, are represented in Figures 5, 6 respectively. In each graph, the dashed line highlights the threshold, set at +43.0°C, as previously defined.

The temperature values obtained from the bipolar catheter in D_1_, with the MED625FLX, independently of the thickness, remained below the threshold (+43.0°C), guarantying safe conditions. Instead, the tissue temperatures with the TPU95A (both 2.5 and 1.0 mm of thickness) at D_1_ Exceeded the threshold, leading to a potential tissue damage. On the other hand, at D_2_ the temperatures were below the threshold for all samples with a range from +29.2°C to +33.0°C, remaining almost stable, close to the control parameters when the RFA was delivered by the bipolar catheter, and with a range between +29.0°C and +37.3°C for unipolar catheter.

**Figure 7:**
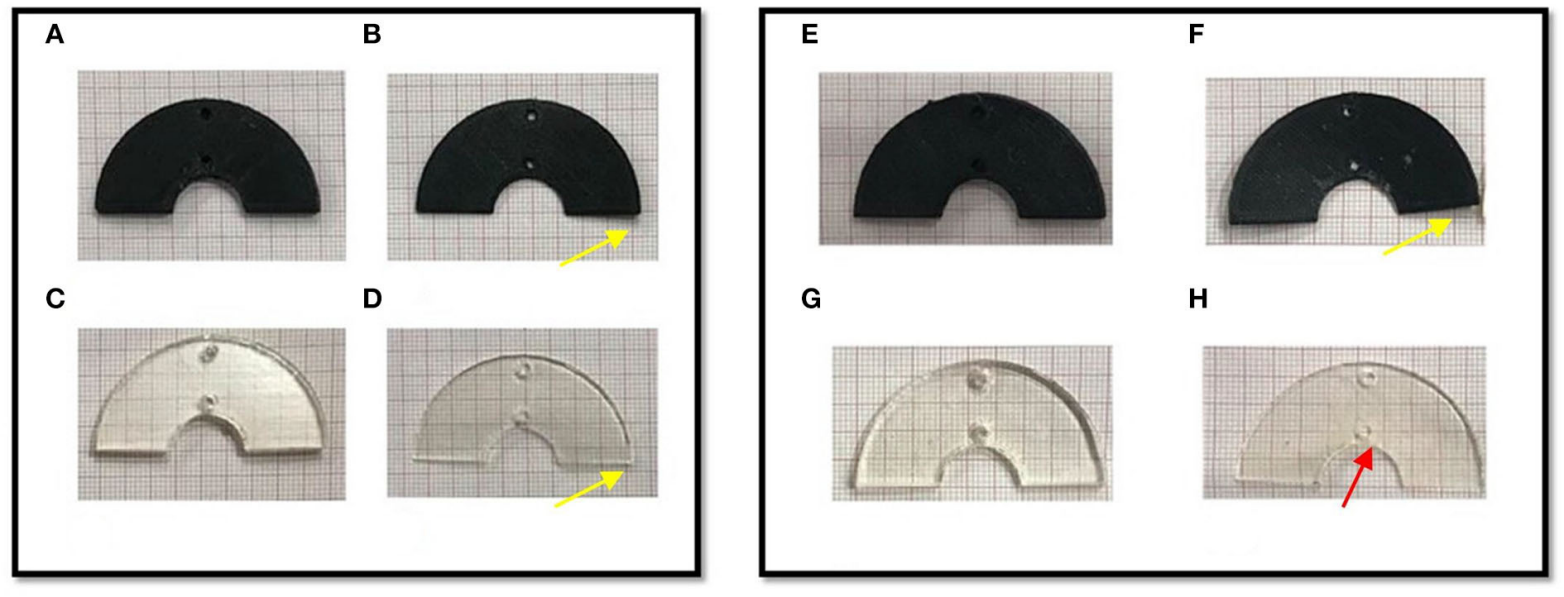
The macroscopically biomaterials changes in geometry after the bi- and uni-polar RFA catheter. The panel on the left shows the biomaterial samples after the bipolar RF ablation, while the panel on the right the biomaterial samples after the unipolar RFA catheter; **(A)** TPU95A of 2.5 mm; **(B)** TPU95A of 1.0 mm; **(C)** MED625FLX of 2.5 mm; **(D)** MED625FLX of 1.0 mm; **(E)** TPU95A of 2.5 mm; **(F)** TPU95A of 1.0 mm; **(G)** MED625FLX of 2.5 mm; **(H)** MED625FLX of 1.0 mm. The yellow arrows indicate the material deflection, the red arrow indicates burn trace.

Finally, the results of geometrical change evaluation are showed in Figure 7. No visible mechanical alterations in MED625FLX and TPU95A 2.5 mm were observed with both ablation catheters. A deflection with RFA application was present, even though without breaking, in MED625FLX and TPU95A of 1.0 mm, resulting in geometrical deformation, which increased with the number of RF applications for both bi- or uni- polar catheters.

## Discussion

Among all materials suitable for medical additive manufacturing, the MED625FLX and TPU95A, due to their mechanical properties, as per material data sheet, could prove to be appropriate candidates, to be used as surgical guide, during RFA treatment (1).

Moreover, biological tests, performed according to the ISO requirements, demonstrated their biocompatibility. Indeed, they have already been used in medical applications as vascular catheters, blood bags, temporary dental use, gingiva masks and other medical tools (2, 5).

However, the behavior of these materials in response to RFA has not been investigated. This is clinically relevant because, if the material becomes warmer, accumulating heat, and reaching +50°C, it can cause cell damage; on the other hand, if the material behaves like a thermal insulant, it could protect the underlying tissue from heating. For a surgical guide, aiming at targeting tissue for RFA, the ideal material should not heat the underlying tissue, leading to unintended damage.

For this reason, the aim of this work was to investigate the thermal properties of MED625FLX and TPU95A, in response to RFA delivered by bipolar and unipolar catheters, analyzing mechanical and geometrical materials changes and measuring temperature propagation in the underlying tissue.

For the purpose of the current study, the tests were performed on PMS of 10.0 mm of thickness; the reason is that the surgical guide is aimed for ventricles and the porcine muscle thickness was similar to human ventricular wall (11). Whereas the biomaterial samples were characterized by semicircular shape, with different thicknesses. This experimental protocol was chosen because it is recognized that the material morphology, including thickness, influences mechanical and thermal properties. Also, baseline tissue temperature parameters were measured, to simulate human body temperature.

Thus, by comparing D_1_ and D_2_, independently of the catheter, the biomaterial and the thickness, the temperatures were inversely proportional to the distance from the ablation source.

Evaluating the temperature results, the last application on the same sample was considered the most crucial, because of the material stress to RFA, thus, the temperatures changes at fourth application were examined. The temperature reached by MED625FLX, independently of the thickness, remained always below the threshold (+43.0°C). Instead, the tissue temperatures with the TPU95A (both 2.5 and 1.0 mm of thickness) at D_1_ exceeded the threshold. Furthermore, focusing on the unipolar RFA response in D_1_, all temperatures suddenly increased, surpassing the threshold, leading to unwanted damages.

Although the thermal conductivity of both materials is low, also the following factors play a role in heat transfer: the gradient of temperature, the length and thickness and the cross-sectional area. In addition to material geometry, other factors conditioning the thermal conductivity of materials include: molecular bonding, structure, and density. Indeed, the units of measure for conductivity must account for the amount of energy transferred per time, thickness (or distance), and temperature difference. Therefore, this study is aimed to assess how the heat transfer in both materials act with 1.0 mm and 2.5 mm thicknesses, evaluating two different RF sources effect at specific distances (13, 14).

Indeed, from the results achieved with bipolar RF in D_1_, both 1.0 mm and 2.5 mm thicknesses of the TPU95A did not seem sufficient to retain the heat, resulting in faster heat transfer to the underlying tissue; the MED625FLX had a variable temperature trend at 1.0 mm, while at 2.5 mm of thickness it had a behavior similar to control conditions, with less heat transfer. From the results with bipolar RF In D_2_, it might be speculated that the higher cross-sectional area decreases the heat transfer, determining lower temperatures.

Focusing on the results with the unipolar RF in D_1_, the heat transfer might have been affected not only by the material thicknesses but also by the punctual energy power of the source.

Moreover, the temperature gradually decreases or increases during continuous RFA because of two phenomena. If the temperatures in the tissue reach high values (>43.0°C), due to a faster heat transmission trough the material, the temperature gradient (between material and tissue) increases. This leads to a homeostatic effect of the material on the temperature, even if the RF source is active. As shown in Figure 5, this happens mostly during unipolar RF at D_1_, for both materials and thicknesses with a behavior opposite respect to the control. On the other hand, if the temperature difference has low values, the inversion of the temperatures slope is due to the continuous transmission of heat by the source. Indeed, the temperature gradient stabilizes in the first 15 s, remaining about constant from 15 s onwards, as shown in Figure 3 for the MED625FX of 2.5 mm of thickness.

Based upon these premises, the only safe combination found is bipolar ablation with MED625FLX.

Concerning the macroscopical changes in materials geometry, a deflection with RFA application was presenting both MED625FLX and TPU95A of 1.0 mm. This was consistent for both bi- and uni- polar catheters.

In conclusion, the TPU95A, independently of thickness, failed almost all thermal tests, reaching high temperatures, that might lead to unintended cell damage. Instead, the 2.5 mm MED625FLX has proved to be a suitable candidate for the cardiac use exclusively during RFA delivered by bipolar catheter, guarantying safe conditions for the underlying tissue, and maintaining unaltered geometrical features. On the other hand, the 1.0 mm MED625FLX, is safe from a thermal point of view, with temperatures below the threshold, but the RFA modified the sample shape during bipolar catheter applications, leading to a possible loss of function.

Lastly, none of these biomaterials are appropriate for unipolar ablation catheter because the temperatures steeply increase, potentially damaging the underlying tissue.

The hereby described 3D printed surgical guides are currently intended only for epicardial use by the surgeon (one ablation catheter on the epicardial surface). Bipolar RFA simulation (with electrodes placed at opposite surfaces) is beyond the scope of this paper but it can be assessed in future studies if a hybrid approach (endo- and epi-cardial ablation) is deemed indicated.

## Conclusions

The use of additive manufacturing has led to benefits in the medical field such as cost reduction, absence of post-assembly, time reduction, patient-specific customization of devices and, possibly, reuse of materials. However, challenges in the clinical application of these medical devices lie in the workflow, namely: patient selection, specific 3D printing, sterilization and intervention.

The 2.5 mm MED625FLX could be used, in the design of surgical guides for RFA bipolar catheter only, because of mechanical, geometrical, and thermal properties. None of biomaterials tested are appropriate for unipolar ablation catheter because of temperature concerns.

The use of MED625FLX is limited to a minimum thickness of 2.5 mm preventing a thinner and more flexible design. Further investigations focusing on optimizing the clinical workflow, on thinner models and bipolar RFA are eagerly awaited.

## Data availability statement

The raw data supporting the conclusions of this article will be made available by the authors, without undue reservation.

## Author contributions

IC, BI, and CA: conception and design of the work. IC, MC, LP, CM, GT, and RR: substantial contributions to the acquisition of data for the work. IC, MC, LP, EB, and GT: substantial contributions to the analysis of data for the work. RR, ML, AG, GC, BI, and CA: substantial contributions to the interpretation of data for the work. IC and MC: drafting the work. GT, RR, ML, AG, GC, BI, and CA: revising the draft of the work critically for important intellectual content. IC, MC, LP, CM, EB, GT, RR, ML, AG, GC, BI, and CA: final approval of the version to be published and agreement to be accountable for all aspects of the work in ensuring that questions related to the accuracy or integrity of any part of the work are appropriately investigated and resolved. All authors contributed to the article and approved the submitted version.

## Conflict of interest

Author ML is consultant for Atricure. GC received compensation for teaching purposes and proctoring from Medtronic, Abbott, Biotronik, Boston Scientific, Acutus Medical. CA receives research grants on behalf of the center from Biotronik, Medtronic, Abbott, LivaNova, Boston Scientific, AtriCure, Philips, and Acutus; received compensation for teaching purposes and proctoring from Medtronic, Abbott, Biotronik, Livanova, Boston Scientific, Atricure, Acutus Medical Daiichi Sankyo. The remaining authors declare that the research was conducted in the absence of any commercial or financial relationships that could be construed as a potential conflict of interest.

## Publisher's note

All claims expressed in this article are solely those of the authors and do not necessarily represent those of their affiliated organizations, or those of the publisher, the editors and the reviewers. Any product that may be evaluated in this article, or claim that may be made by its manufacturer, is not guaranteed or endorsed by the publisher.
