# Supplementary material for: Temperature analysis of 3D-printed biomaterials during unipolar and bipolar radiofrequency ablation procedure
Source: Front Cardiovasc Med. 2022 Sep 14;9:978333. doi: 10.3389/fcvm.2022.978333 (PMC9515363; doi:10.3389/fcvm.2022.978333)
Supplement: Supplementary file 1 [file Table_1.DOCX]

# SUpplemantary materials

## Table S 1. Statistical analysis of temperatures means between control and PMS under the biomaterial samples in D_1_

| ***Bipolar Catheter*** | | | | | |
| --- | --- | --- | --- | --- | --- |
| ***Distance*** | ***Time*** | ***Biomaterial*** | ***Control Mean (SD)*** | ***Biomaterial Mean (SD)*** | ***p-value*** |
| D_1_ | 0 s | Med_2.5mm | 31.4 (2.8) | 33.1 (1.4) | 0.314 |
|  | 10 s | Med_2.5mm | 31.7 (2.7) | 33.9 (1.6) | 0.210 |
|  | 15 s | Med_2.5mm | 31.8 (2.7) | 34.1 (1.5) | 0.192 |
|  | 30 s | Med_2.5mm | 32.2 (2.7) | 34.8 (1.6) | 0.139 |
|  | 0 s | Med_1.0mm | 31.4 (2.8) | 34.2 (2.4) | 0.186 |
|  | 10 s | Med_1.0mm | 31.7 (2.7) | 34.9 (3.4) | 0.191 |
|  | 15 s | Med_1.0mm | 31.8 (2.7) | 35.1 (3.1) | 0.159 |
|  | 30 s | Med_1.0mm | 32.2 (2.7) | 35.1 (2.2) | 0.141 |
|  | 0 s | Tpu_2.5mm | 31.4 (2.8) | 36.2 (5.7) | 0.180 |
|  | 10 s | Tpu_2.5mm | 31.7 (2.7) | 37.1 (5.3) | 0.122 |
|  | 15 s | Tpu_2.5mm | 31.8 (2.7) | 36.9 (4.9) | 0.119 |
|  | 30 s | Tpu_2.5mm | 32.2 (2.7) | 36.9 (4.2) | 0.107 |
|  | 0 s | Tpu_1.0mm | 31.4 (2.8) | 38.4 (8.1) | 0.155 |
|  | 10 s | Tpu_1.0mm | 31.7 (2.7) | 38.1 (7.1) | 0.142 |
|  | 15 s | Tpu_1.0mm | 31.8 (2.7) | 38.0 (6.7) | 0.138 |
|  | 30 s | Tpu_1.0mm | 32.2 (2.7) | 37.6 (5.6) | 0.128 |

For the p-value computation different thicknesses of biomaterials have been considered. The SD is the standard deviation of temperature measurements.

## Table S 2. Statistical analysis of temperatures means between control and PMS under the biomaterial samples in D_2_

| ***Bipolar Catheter*** | | | | | |
| --- | --- | --- | --- | --- | --- |
| ***Distance*** | ***Time*** | ***Biomaterial*** | ***Control Mean (SD)*** | ***Biomaterial Mean (SD)*** | ***p-value*** |
| D_2_ | 0 s | Med_2.5mm | 29.2 (1.9) | 29.7 (0.5) | 0.626 |
|  | 10 s | Med_2.5mm | 29.5 (1.8) | 29.7 (0.6) | 0.816 |
|  | 15 s | Med_2.5mm | 29.6 (1.8) | 29.8 (0.6) | 0.838 |
|  | 30 s | Med_2.5mm | 29.8 (1.9) | 29.9 (0.7) | 0.905 |
|  | 0 s | Med_1.0 mm | 29.2 (1.9) | 32.0 (0.6) | 0.037 |
|  | 10 s | Med_1.0 mm | 29.5 (1.8) | 32.0 (0.7) | 0.040 |
|  | 15 s | Med_1.0 mm | 29.6 (1.8) | 32.0 (0.7) | 0.046 |
|  | 30 s | Med_1.0 mm | 29.8 (1.9) | 32.0 (0.6) | 0.063 |
|  | 0 s | Tpu_2.5mm | 29.2 (1.9) | 29.8 (1.2) | 0.661 |
|  | 10 s | Tpu_2.5mm | 29.5 (1.8) | 29.7 (1.3) | 0.843 |
|  | 15 s | Tpu_2.5mm | 29.6 (1.8) | 29.8 (1.2) | 0.839 |
|  | 30 s | Tpu_2.5mm | 29.8 (1.9) | 30.1 (1.2) | 0.764 |
|  | 0 s | Tpu_1.0mm | 29.2 (1.9) | 32.3 (0.4) | 0.024 |
|  | 10 s | Tpu_1.0mm | 29.5 (1.8) | 32.0 (0.2) | 0.029 |
|  | 15 s | Tpu_1.0mm | 29.6 (1.8) | 32.0 (0.2) | 0.036 |
|  | 30 s | Tpu_1.0mm | 29.8 (1.9) | 31.9 (0.3) | 0.066 |

For the p-value computation different thicknesses of biomaterials have been considered. The SD is the standard deviation of temperature measurements.

## Table S 3. Statistical analysis of temperatures means between control and PMS under the biomaterial samples in D_1_

| ***Unipolar Catheter*** | | | | | |
| --- | --- | --- | --- | --- | --- |
| ***Distance*** | ***Time*** | ***Biomaterial*** | ***Control Mean (SD)*** | ***Biomaterial Mean (SD)*** | ***p-value*** |
| D_1_ | 0 s | Med_2.5 mm | 34.7 (1.7) | 59.1 (14.1) | 0.014 |
|  | 10 s | Med_2.5 mm | 36.3 (2.1) | 50.6 (9.9) | 0.030 |
|  | 15 s | Med_2.5 mm | 36.6 (2.1) | 49.5 (7.3) | 0.014 |
|  | 30 s | Med_2.5 mm | 36.9 (1.8) | 45.9 (5.0) | 0.016 |
|  | 60 s | Med_2.5 mm | 36.2 (1.4) | 41.6 (3.0) | 0.083 |
|  | 0 s | Med_1.0 mm | 34.7 (1.7) | 50.2 (16.4) | 0.107 |
|  | 10 s | Med_1.0 mm | 36.3 (2.1) | 44.6 (9.0) | 0.123 |
|  | 15 s | Med_1.0 mm | 36.6 (2.1) | 43.5 (7.3) | 0.120 |
|  | 30 s | Med_1.0 mm | 36.9 (1.8) | 41.7 (5.0) | 0.126 |
|  | 60 s | Med_1.0 mm | 36.2 (1.4) | 39.9 (2.7) | 0.155 |
|  | 0 s | Tpu_2.5 mm | 34.7 (1.7) | 64.8 (6.3) | < 0.001 |
|  | 10 s | Tpu_2.5 mm | 36.3 (2.1) | 55.8 (2.4) | < 0.001 |
|  | 15 s | Tpu_2.5 mm | 36.6 (2.1) | 54.0 (2.0) | < 0.001 |
|  | 30 s | Tpu_2.5 mm | 36.9 (1.8) | 50.1 (1.4) | < 0.001 |
|  | 60 s | Tpu_2.5 mm | 36.2 (1.4) | 44.5 (0.8) | < 0.001 |
|  | 0 s | Tpu_1.0 mm | 34.7 (1.7) | 56.0 (17.3) | 0.050 |
|  | 10 s | Tpu_1.0 mm | 36.3 (2.1) | 51.5 (13.0) | 0.061 |
|  | 15 s | Tpu_1.0 mm | 36.6 (2.1) | 50.2 (11.0) | 0.052 |
|  | 30 s | Tpu_1.0 mm | 36.9 (1.8) | 47.2 (7.8) | 0.041 |
|  | 60 s | Tpu_1.0 mm | 36.2 (1.4) | 43.6 (3.9) | 0.070 |

For the p-value computation different thicknesses of biomaterials have been considered. The SD is the standard deviation of temperature measurements.

**Table S 4. Statistical analysis of temperatures means between control and PMS under the biomaterial samples in D_2_**

| ***Unipolar Catheter*** | | | | | |
| --- | --- | --- | --- | --- | --- |
| ***Distance*** | ***Time*** | ***Biomaterial*** | ***Control Mean (SD)*** | ***Biomaterial Mean (SD)*** | ***p-value*** |
| D_2_ | 0 s | Med_2.5 mm | 31.1 (1.0) | 33.6 (1.5) | 0.030 |
|  | 10 s | Med_2.5 mm | 31.7 (1.3) | 32.5 (0.6) | 0.351 |
|  | 15 s | Med_2.5 mm | 32.7 (1.7) | 32.1 (0.7) | 0.568 |
|  | 30 s | Med_2.5 mm | 33.2 (2.2) | 31.8 (1.1) | 0.317 |
|  | 60 s | Med_2.5 mm | 34.8 (2.9) | 31.9 (1.4) | 0.156 |
|  | 0 s | Med_1.0 mm | 31.1 (1.0) | 35.1 (1.6) | 0.005 |
|  | 10 s | Med_1.0 mm | 31.7 (1.3) | 34.6 (1.5) | 0.046 |
|  | 15 s | Med_1.0 mm | 32.7 (1.7) | 34.4 (1.4) | 0.174 |
|  | 30 s | Med_1.0 mm | 33.2 (2.2) | 34.5 (1.4) | 0.362 |
|  | 60 s | Med_1.0 mm | 34.8 (2.9) | 34.6 (1.4) | 0.921 |
|  | 0 s | Tpu_2.5 mm | 31.1 (1.0) | 36.3 (6.7) | 0.176 |
|  | 10 s | Tpu_2.5 mm | 31.7 (1.3) | 33.8 (3.0) | 0.329 |
|  | 15 s | Tpu_2.5 mm | 32.7 (1.7) | 33.1 (3.2) | 0.812 |
|  | 30 s | Tpu_2.5 mm | 33.2 (2.2) | 32.6 (2.4) | 0.760 |
|  | 60 s | Tpu_2.5 mm | 34.8 (2.9) | 32.8 (1.9) | 0.356 |
|  | 0 s | Tpu_1.0 mm | 31.1 (1.0) | 33.9 (3.1) | 0.137 |
|  | 10 s | Tpu_1.0 mm | 31.7 (1.3) | 34.2 (2.7) | 0.198 |
|  | 15 s | Tpu_1.0 mm | 32.7 (1.7) | 34.5 (2.9) | 0.300 |
|  | 30 s | Tpu_1.0 mm | 33.2 (2.2) | 35.2 (2.8) | 0.307 |
|  | 60 s | Tpu_1.0 mm | 34.8 (2.9) | 35.9 (2.3) | 0.632 |

For the p-value computation different thicknesses of biomaterials have been considered. The SD is the standard deviation of temperature measurements.
